# QUALITY OF LIFE IN PATIENTS WITH ROTATOR CUFF ARTHROPATHY

**DOI:** 10.1590/1413-785220172506173893

**Published:** 2017

**Authors:** ARNALDO AMADO FERREIRA, EDUARDO ANGELI MALAVOLTA, JORGE HENRIQUE ASSUNÇÃO, MAURO EMILIO CONFORTO GRACITELLI, GUILHERME PEREIRA OCAMPOS, EVELINDA MARRAMON TRINDADE

**Affiliations:** 1. Instituto de Ortopedia e Traumatologia, Hospital das Clínicas, Faculdade de Medicina da Universidade de São Paulo, São Paulo, SP, Brazil.; 2. São Paulo State Health Technology Assessment Network, São Paul State Department of Health, São Paulo, SP, Brazil.

**Keywords:** Arthroplasty, replacement. Joint diseases. Osteoarthritis. Rotator cuff. Quality of life., Artroplastia de substituição, Artropatias, Osteoartrose, Manguito rotador, Qualidade de vida.

## Abstract

**Objective:**

To compare quality of life (according to the SF-12) in patients with rotator cuff arthropathy with controls paired by sex and age. Secondary objectives are to compare the groups according to the ASES and VAS scales.

**Methods:**

This cross-sectional study with controls paired by sex and age compared patients with rotator cuff arthropathy with surgical indication for reverse shoulder arthroplasty. The groups were compared according to the SF-12, ASES, and VAS scales.

**Results:**

The groups consisted of 38 individuals, 28 women. The SF-12 demonstrated a significant difference in the physical component, with the cases scoring 31.61 ± 6.15 and the controls 49.39 ± 6.37 (p<0.001). For the mental component, the difference was not significant, with the cases scoring 44.82 ± 13.18 and the controls 48.96 ± 8.65 (p=0.109). The cases scored 7.34 ± 2.11 on the VAS and 31.26 ± 15.12 on the ASES, while the controls scored 0.55 ± 1.31 and 97.53 ± 6.22, respectively (p<0.001).

**Conclusion:**

Patients with rotator cuff arthropathy had poorer results for the physical component of the SF-12 than the controls. They also had poorer functional results according to the ASES scale, and more pain according to the VAS. **Level of Evidence III, Case Control Study.**

## RESUMO

**Objetivo:**

Comparar a qualidade de vida, de acordo com o SF-12, entre pacientes com artropatia do manguito rotador e controles pareados por sexo e idade. É objetivo secundário a comparação dos grupos de acordo com as escalas ASES e EVA.

**Métodos:**

Estudo transversal com controles pareados por sexo e idade, que comparou pacientes com artropatia do manguito rotador e indicação de artroplastia reversa do ombro com indivíduos sadios. Os grupos foram comparados quanto às escalas SF-12, ASES e EVA.

**Resultados:**

Os grupos foram formados por 38 indivíduos, sendo 28 do sexo feminino. O SF-12 apresentou diferença significativa no componente físico, tendo os casos registrado 31,61 ± 6,15 e os controles 49,39 ± 6,37 (p < 0,001). Para o componente mental, a diferença não foi significativa, tendo os casos apresentado 44,82 ± 13,18 e os controles 48,96 ± 8,65 (p=0.109). Os casos apresentaram EVA de 7,34 ± 2,11 e ASES de 31,26 ± 15,12, enquanto os controles apresentaram 0,55 ± 1,31 e 97,53 ± 6,22, respectivamente (p < 0,001).

**Conclusão:**

Os pacientes com artropatia do manguito rotador apresentam piores resultados no componente físico do SF-12 quando comparados aos controles. Têm, ainda, piores resultados funcionais pela escala da ASES e mais dor pela EVA. **Nível de Evidência III, Estudo de Caso-Controle.**

## INTRODUCTION

Arthropathy of the rotator cuff is arthritis of the glenohumeral joint associated with massive rupture of the rotator cuff.^1^ This injury affects 2.5% of the population over age 70^2^ and can lead to pain and significant functional limitations.^1^

Scales that assess quality of life (QOL) are often used in studies of osteoarthritis. The significant impact this condition has on patient QOL has already been described for osteoarthritis of the knee,^3^ ^-^ ^6^ the hip,^3^ ^,^ ^5^ ^,^ ^6^ and the hand.^6^ ^,^ ^7^

Few studies evaluate the effect of reverse arthroplasty of the shoulder on patient QOL, whether this procedure treats degenerative injury^8^ or fractures.^9^ However, no study as of this time has compared QOL in patients with arthropathy of the rotator cuff with that of a control group.

The primary objective of this study is to compare QOL as measured by the Short Form 12 Health Survey (SF-12)^10^ between patients with rotator cuff arthropathy with indication of reverse arthroplasty and controls matched for sex and age. Secondary objectives are to compare the groups according to function and pain, using the scales from the American Shoulder and Elbow Surgeons Standardized Shoulder Assessment Form (ACES)^11^ and the visual analog scale for pain (VAS).

## MATERIALS AND METHODS

We conducted a cross-sectional study with matched controls at a 1:1 ratio. At our institution’s outpatient clinic, we assessed patients with a diagnosis of arthropathy of the rotator cuff and indication for reverse arthroplasty of the shoulder, with respect to their QOL. The control group was composed of people accompanying patients to the same outpatient clinic, who were matched by sex and age. An age difference of ±2 years was tolerated.

The patients were assessed between July 21, 2015 and April 13, 2016. The study was approved by the institutional review board under process number 1103 and did not receive any type of funding.

### The criteria for indication of reverse arthroplasty were:

- Diagnosis of arthropathy of the rotator cuff
- Active elevation of less than 90°
- Unsuccessful non-surgical treatment performed for at least 6 months.

### After the informed consent form was signed, information on the following variables was collected in an interview:

- The quality of life scale from the Short Form 12 Health Survey (SF-12),^10^ considering the primary outcome;
- Functional scale from the American Shoulder and Elbow Surgeons Standardized Shoulder Assessment Form (ASES)^11^ and visual analog pain scale (VAS), secondary outcomes;
- Demographic data: sex, age, clinical comorbidities (cardiovascular, pulmonary, neurological, rheumatic, urological, endocrine, psychiatric, immunological, and neoplastic disorders), orthopedic comorbidities (spine, shoulder and elbow, wrist and hand, hip, knee, foot and ankle), body mass index (BMI), and number of medications taken daily;
- Presence or absence of pain in the shoulder, and duration of symptoms.

### Calculation of the sample

The minimal clinically important difference (MCID) on the SF-12 has not yet been established for rotator cuff arthropathy. In a study on total knee arthroplasty, the MCID was determined to be 4.8 points for the physical component of the SF-12, with a standard deviation of 10.4.^12^ In a conservative scenario, considering a MCID of 10 points and a standard deviation of 15, we would need 36 individuals in each group.

### Statistical analysis

We assessed the normality of the continuous variables using the Kolmogorov-Smirnov test, and homogeneity using the Levene test. The continuous quantitative variables were expressed as means and standard deviation, while the categorical variables were expressed as absolute values and percentages.

The comparison between the cases and controls with respect to the different variables was performed using the chi-squared or Fisher’s exact test for the categorical variables. For the continuous variables, this comparison was assessed using the non-paired Student’s t test if the data was parametrically distributed, or the Wilcoxon test for non-nonparametric distribution.

We used SPSS version 20.0 software (SPSS Inc, Chicago, Illinois, USA) for the data analysis, and a significance level of 5%.

## RESULTS

The institution’s database included 45 patients recommended for reverse arthroplasty of the shoulder. Five of these could not be located, one had died, and one did not agree to take part in the study. Consequently, 38 patients were interviewed to comprise the case group cases, and an equal number were selected as controls. The two groups had 28 women.

The cases had involvement of the right side in 68.4% (26/38), the left side in 23.7% (9/38), and bilateral involvement in 7.9% (3/38). The mean time they experienced symptoms was 128.97 months.

The groups did not differ significantly in age, BMI, or previous surgeries in sites other than the shoulder (p=0.878, p=0.159 and p=0.489, respectively). The comorbidities, excluding rheumatic diseases (p=0.028), also showed no differences between the groups. The patients recommended for reverse arthroplasty used a significantly larger number of medications (p=0.021). With respect to orthopedic diseases, the cases had significantly more shoulder symptoms (p<0.001) and no significant difference in other locations. The general characteristics of the sample can be seen in Table 1.

**Table 1:** General characteristics of the sample.

	Cases	Controls	p
Sex			
Men	10	10	>0.999
Women	28	28	
Age (years)	67.34 ± 8.02	67.05 ± 8.33	0.878
Body mass index	28.10 ± 5.65	26.54 ± 3.66	0.159
**Comorbidities**			
Hypertension	28	27	0.798
Cardiovascular diseases	1	3	0.615
Rheumatic diseases	8	1	0.028
Lung diseases	1	0	>0.999
Neurological diseases	3	1	0,615
Urological diseases	0	0	>0.999
Diabetes mellitus	5	7	0.754
Hypercholesterolemia	6	4	0.736
Hypothyroidism	6	2	0.262
Psychiatric diseases	6	5	>0.999
Immunologic diseases	0	0	>0.999
Neoplastic diseases	0	1	>0.999
Number of medications per day/patient	4.47 ± 3.28	2.87 ± 2.63	0.021
Previous orthopedic surgeries (other than shoulder)	23	19	0.489
Number of patients with previous shoulder surgeries	13	1	<0.001
**Orthopedic diseases**			
Spine	4	6	0.736
Shoulder	38	5	<0.001
Hand and wrist	3	1	0.615
Hip	4	0	0,115
Knee	11	4	0.082
Foot and ankle	3	1	0.615

The SF-12 showed a significant difference in the physical component, with the cases scoring 31.61 ± 6.15 and the controls 49.39 ± 6.37 (p<0.001). For the mental component, the difference was not significant, with the cases scoring 44.82 ± 13.18 and the controls 48.96 ± 8.65 (p<0.109). The cases presented a VAS score of 7.34 ± 2.11, and ASES score of 31.26 ± 15.12, while the control scores were 0.55 ± 1.31 and 97.53 ± 6.22, respectively (p<0.001). The data can be seen in Table 2.

**Table 2:** Outcomes.

	Cases	Controls	p
VAS	7.34 ± 2.11	0.55 ± 1.31	<0.001
ASES	31.26 ± 15.12	97.53 ± 6.22	<0.001
SF-12 Physical	31.61 ± 6.15	49.39 ± 6.37	<0.001
SF-12 Mental	44.82 ± 13.18	48.96 ± 8.65	0.109

VAS: Visual Analog Pain Scale; ASES: Functional scale of the American Shoulder and Elbow Surgeons Standardized Shoulder Assessment Form; SF-12: Quality of life scale of the Short Form 12 Health Survey.

## DISCUSSION

Arthropathy of the rotator cuff affects approximately 4% of patients with rotator cuff tears.^13^ The clinical manifestations of this disease are variable, and patient symptoms may be minimal with satisfactory function or range up to pseudoparalysis.^1^ The treatment of rotator cuff arthropathy represents a challenge to orthopedists, but reverse arthroplasty is a viable solution that can improve QOL.^8^

In a systematic review of the indications for reverse arthroplasty of the shoulder, Smith et al.^14^ found that painful pseudoparalysis is the main reason to perform surgery. Analyzes of QOL in indicating surgery are not cited in this or other studies.^8^ ^,^ ^14^ ^,^ ^15^ We believe that QOL should be evaluated before and after arthroplasty, and should be part of the clinical reasoning at the time surgery is recommended. Many patients with this condition are elderly, with comorbid conditions and limitations inherent to age.^16^

Our results demonstrate that patients with a diagnosis of rotator cuff arthropathy and indication for reverse arthroplasty show significant impact on the physical component of QOL according to the SF-12, when compared to controls adjusted for sex and age. Sick individuals tend to have poorer outcomes on the mental component of this scale. Several studies have demonstrated the impact of osteoarthritis of the knee,^3^ ^-^ ^6^ the hip,^3^ ^,^ ^5^ ^,^ ^6^ and the hand^6^ ^,^ ^7^ in QOL, comparing sick individuals with healthy controls. However, these studies do not evaluate the involvement of the glenohumeral joint, whether by primary osteoarthrosis or arthropathy of the rotator cuff. The predominant impact on the physical component of QOL has been observed by other authors.^5^ ^,^ ^6^ Our study also stressed that the arthropathy of the rotator cuff significantly affects shoulder pain and function when these are analyzed by the VAS and ASES scale. The other studies comparing patients with osteoarthritis in other joints with healthy controls do not assess these outcomes.^3^ ^-^ ^7^ ^,^ ^17^

Castricini et al.,^8^ in a study on the use of reverse arthroplasty of the shoulder in degenerative disorders (arthropathy of the rotator cuff, irreparable rupture of the rotator cuff, and primary glenohumeral arthritis), noted that the procedure provides QOL similar to that of the healthy population. Mangano et al.^15^ found similar results, studying only elderly patients. In a study on the use of reverse arthroplasty in proximal fractures of the humerus, Lopiz et al.^9^ also observed final QOL outcomes comparable to the unaffected population. However, these articles do not detail the preoperative QOL values, like the other case series evaluated.^18^ ^-^ ^22^ As of this writing, this present study is the first to compare QOL, function, and pain in patients with a diagnosis of rotator cuff arthropathy and an unaffected population.

The groups, although they were only paired for sex and age, showed a similar distribution for most of the analyzed variables, reinforcing the validity of the inclusion criteria. It should be emphasized that the cases consumed significantly more medications than the controls, which increases the risk of side effects and drug interactions. Furthermore, the groups showed no difference in relation to BMI. Excess weight can be a confounding factor in the analysis, since obesity negatively affects QOL.^23^ Some authors of studies that evaluated QOL in patients with osteoarthritis of the legs did not mention this variable,^4^ ^,^ ^5^ while others found that the arthritis group had a higher BMI.^6^ Moreover, the tool we used to assess QOL, the SF-12, is self-applied, validated, and its results are comparable to the SF-36.^10^

This study has limitations. The sample consisted of patients with surgical indication for reverse arthroplasty for rotator cuff arthroplasty, representing only the symptomatic cases of this disease that did not improve after conservative treatment. Patients with rotator cuff arthropathy may present few symptoms and satisfactory shoulder function.^1^ The absence of a group of oligosymptomatic patients with rotator cuff arthropathy is the main limitation of our study. Although the sample was small, it was sufficient to prove our hypothesis, given the significant difference we found. Furthermore, the cross-sectional design did not allow us to evaluate temporal variations in the outcomes and we did not analyze pre- and postoperative pain.

## CONCLUSION

Patients with rotator cuff arthropathy who were recommended for reverse arthroplasty have poorer results for the physical component of the SF-12 when compared to controls. They also had poorer functional outcomes as measured by the ASES scale and more pain as measured by the VAS.
